# Molecular and clinical study of a cohort of 110 Algerian patients with autosomal recessive ataxia

**DOI:** 10.1186/s12881-015-0180-3

**Published:** 2015-06-12

**Authors:** Wahiba Hamza, Lamia Ali Pacha, Tarik Hamadouche, Jean Muller, Nathalie Drouot, Farida Ferrat, Samira Makri, Malika Chaouch, Meriem Tazir, Michel Koenig, Traki Benhassine

**Affiliations:** 1 Laboratoire de Biologie Cellulaire et Moléculaire, Faculté des Sciences Biologiques, USTHB, Alger, Algeria; 2 Service de Neurologie, CHU Mustapha Bacha, Alger, Algeria; 3 Laboratoire de Neurosciences, Université d’Alger 1, Alger, Algeria; 4 Laboratoire de Biologie Moléculaire, Faculté des Sciences, UMBB, Boumerdes, Algeria; 5 Institut de Génétique et de Biologie Moléculaire et Cellulaire, CNRS/Université de Strasbourg UMR7104, INSERM U964, Illkirch, France; 6 Laboratoire de Diagnostic Génétique, Hôpitaux Universitaires de Strasbourg, Strasbourg, France; 7 Service de Neurologie, CHU Ben Aknoun, Alger, Algeria; 8 Service de Neurologie, EHS Ali Aït Idir, Alger, Algeria; 9 Laboratoire de Génétique de Maladies Rares, Institut Universitaire de Recherche Clinique, Université de Montpellier, CHU de Montpellier, Montpellier, France

**Keywords:** ARCA, Autosomal recessive ataxias, Algeria, Cohort, Molecular analysis

## Abstract

**Background:**

Autosomal recessive cerebellar ataxias (ARCA) are a complex group of neurodegenerative disorders with great genetic and phenotypic heterogeneity, over 30 genes/loci have been associated with more than 20 different clinical forms of ARCA. Genetic heterogeneity combined with highly variable clinical expression of the cerebellar symptoms and overlapping features complicate furthermore the etiological diagnosis of ARCA. The determination of the most frequent mutations and corresponding ataxias, as well as particular features specific to a population, are mandatory to facilitate and speed up the diagnosis process, especially when an appropriate treatment is available.

**Methods:**

We explored 166 patients (115 families) refered to the neurology units of Algiers central hospitals (Algeria) with a cerebellar ataxia phenotype segregating as an autosomal recessive pattern of inheritance. Genomic DNA was extracted from peripheral blood samples and mutational screening was performed by PCR and direct sequencing or by targeted genomic capture and massive parallel sequencing of 57 genes associated with inherited cerebellar ataxia phenotypes.

**Results:**

In this work we report the clinical and molecular results obtained on a large cohort of Algerian patients (110 patients/76 families) with genetically determined autosomal recessive ataxia, representing 9 different types of ARCA and 23 different mutations, including 6 novel ones. The five most common ARCA in this cohort were Friedreich ataxia, ataxia with isolated vitamin E deficiency, ataxia with oculomotor apraxia type 2, autosomal recessive spastic ataxia of Charlevoix-Saguenay and ataxia with oculomotor apraxia type 1.

**Conclusion:**

We report here a large cohort of patients with genetically determined autosomal recessive ataxia and the first study of the genetic context of ARCA in Algeria. This study showed that in Algerian patients, the two most common types of ataxia (Friedreich ataxia and ataxia with isolated vitamin E deficiency) coexist with forms that may be less common or underdiagnosed. To refine the genotype/phenotype correlation in rare and heteregeneous diseases as autosomal recessive ataxias, more extensive epidemiological investigations and reports are necessary as well as more accurate and detailed clinical characterizations. The use of standardized clinical and molecular protocols would thus enable a better knowledge of the different forms of ARCA.

## Background

Inherited cerebellar ataxia is the consequence of an impairment of the cerebellum, the brainstem and/or spinocerebellar tracts. Phenotypic manifestations may include gait-limb ataxia, frequent falls, dysarthria, adiadochokinesia, ophthalmological abnormalities and may also involve other neurological and/or extraneurological symptoms. Progression of these features lead to the loss of ambulatory abilities and often to an early death [1, 2].

Although all patterns of inheritance have been described in hereditary cerebellar ataxia [2, 3], we focused in this work on the Autosomal Recessive Cerebellar Ataxias “ARCA” forms because of the consanguineous context characterizing the Algerian population.

ARCA’s first symptoms usually appear before 25 years old, despite the fact that late-onset forms have been described [1], and are considered as one of the most complex group in neurogenetics with more than 20 different clinical entities and at least 30 associated genes/loci [4]. Some of these genes are responsible for worldwide well-known ataxia forms, while others underlie very rare forms. Among these, some have only been described in an isolated population or even in one family [4, 5].

Despite the increasing knowledge in the molecular aspects of ARCA, a significant proportion of patients remain with unidentified ARCA. In addition to the genetic heterogeneity of this group of ataxias, there is an important phenotypic variability in the expression of the cerebellar impairment. The clinical manifestations are indeed as varied as the possible underlying mutations. The heterogeneous expression of these disorders occurs within unrelated patients as well as between siblings of the same family and with the same ataxia form. Furthermore, the existence of atypical phenotypes and overlapping characteristics bring more complexity to the etiological diagnosis of ARCA. The delineation of the specific clinical features to each ARCA is in fact under permanent discussion, thus complicating the molecular investigations, but also make it an absolute necessity.

Despite ARCA’s high physiopathological diversity, some clinical entities are known to be the most frequent, such as Friedreich ataxia (FRDA) and ataxia telangiectasia (AT) in Europe [6–8] or FRDA and AVED in the Algerian population [9].

Given the great clinical and genotypic heterogeneity, as well as overlapping features, identification of the most common mutations and corresponding ataxia forms, together with the specific clinical features observed in a particular population, is crucial to facilitate and speed up the diagnosis process, especially when an appropriate treatment could be provided.

This work reports the molecular findings and phenotypic features of a large cohort of 110 Algerians patients with autosomal recessive cerebellar ataxia representing 9 different ataxia entities. We also report some novel mutations, additionaly to mutations already described so far.

## Methods

### Patients selection

We studied 166 patients (115 families) refered to the neurology units of Algiers central hospitals (Algeria) and report a cohort of 110 Algerian patients, belonging to 76 families, with identified causal mutations.

All patients studied had cerebellar ataxia phenotypes with an autosomal recessive pattern of inheritance. The existence of similar cases among siblings (and/or family members) were observed in 30 families and a consanguineous background were noted for about 80 % of the families. Whenever possible, affected siblings and other family members were also analyzed.

Blood samples were obtained for all the patients who gave informed consent to take part in the study which was approved by the Ministry of Health, Health Ethic Committee, Algeria.

### Molecular analysis

Genomic DNA was extracted from peripheral blood samples using QIAamp DNA Mini Kit®.

For all 166 patients, the approach consisted in performing a systematic screening, using PCR and direct sequencing, of the mutations responsible for the major forms of ataxia: the (GAA)_n_ expansion in the first intron of the frataxin gene causing Friedreich ataxia, and the c.744delA mutation in exon 5 of the *TTPA* gene which causes ataxia with isolated vitamin E deficiency.

When these targeted mutations were absent, patients’ DNA was then analyzed by PCR and sequencing of genes accountable for some other forms of ataxia (Ataxia with ocular apraxia 1 and 2 “AOA1, AOA2”; Autosomal recessive spastic ataxia of Charlevoix-Saguenay “ARSACS”; Polyneuropathy, hearing loss, ataxia, retinitis pigmentosa, and cataract syndrome “PHARC”; and Autosomal recessive cerebellar ataxia 2 “ARCA2”) by taking into account the specific clinical indications, while not always strictly relying on them because of the encountered phenotypic heterogeneity, as well as on the basis of previous reports incriminating these genes in our population.

Finally, taking advantage of the availability of targeted genomic capture of 57 genes (responsible for inherited cerebellar ataxia phenotypes as well as genes associated with pathologies presenting ataxia as a major and/or only sign) and massive parallel sequencing using the Agilent SureSelect kit®, we were able to select 16 of our patients, taking into account specific clinical indications as well as more heterogeneous phenotypes. The used strategy was similar to the one described by Vasli *et al.* [10].

## Results

### Clinical characteristics

Out of 110 patients with identified molecular alterations, 68 had undergone a complete neurologic examination. Those patients belonged to 52 unrelated families among which 40 families (55 patients out of 68) were consanguineous. 57 patients were males and 53 females. The sex ratio was not statistically different (1.07). The mean age at onset for those 68 patients was 11.1 ± 5.97 SD years and ranged from birth to 35 years old.

The most common onset sign was gait limb ataxia with unbalance and lack of coordination. Cerebellar syndrome was a constant feature, although with various degrees of disability. Most patients had ataxia (unbalance and limb incoordination) and experienced frequent falls along with different symptoms like dysmetria and adiadochokinesia. Different stages of disability were observed: most patients were capable of walking without help, some of them with unilateral or bilateral help, while 3 were wheelchair-bound at the time of clinical diagnosis. A few patients had a mild cerebellar syndrome. Table 1 summarizes the clinical data of this cohort of 68 patients. MRI results were available for 50 out of 68 patients and EMG examination was performed in 51 patients. Results are summarized in Table 2.

**Table 1:** Clinical features of a group of Algerian patients with autosomal recessive ataxia

Features (68 patients)	Number of patients (%) or value
Age of onset (mean, range-years)	11.1 ± 5.97 SD (birth-35)
Cerebellar syndrome	68 (100 %)
Onset sign	
Gait ataxia	47 (69.12 %)
Gait ataxia, frequent falls	7 (10.29 %)
Gait ataxia, muscle weakness	6 (8.82 %)
Gait ataxia, dysarthria	3 (4.41 %)
Gait ataxia, head tremor	1 (1.47 %)
Head tremor	1 (1.47 %)
Head tremor, dysarthria	1 (1.47 %)
Psychomotor retardation	2 (2.94 %)
Dysarthria	59 (86.76 %)
Nystagmus	37 (54.41 %)
Visual acuity decline	7 (10.29 %)
Spasticity	11 (16.17 %)
Babinski sign	22 (32.35 %)
Dysmophric signs	
Scoliosis	11 (16.17 %)
Pes cavus	20 (29.41 %)
Scoliosis, pes cavus	21 (30.88 %)
Scoliosis, flat feet	3 (4.41 %)
Pes cavus, cranio-facial dysmorphy	1 (1.47 %)
None	12 (17.64 %)
Cognitive impairment	6 (8.82 %)
Cardiomyopathy	7 (10.3 %)
Epilepsy (epileptic seizures)	3 (4.41 %)
Oculomotor apraxia	5 (7.35 %)
Hypoacusia	6 (8.82 %)
Head tremor	10 (14.7 %)

**Table 2:** Brain MRI and EMG results of a group of Algerian patients with autosomal recessive ataxia

Imaging and elctrophysiological investigation	Number of patients (%)
Brain MRI (50 patients)	
Normal	21 (30.88 %)
Cerebellar hemisphere atrophy	14 (20.6 %)
Cerebellar and vermian atrophy	7 (10.3 %)
Vermian atrophy	5 (7.35 %)
Frontoparietal atrophy	1 (1.47 %)
Parietooccipital atrophy	1 (1.47 %)
Spinal cord atrophy	1 (1.47 %)
Unavailable	18 (26.47 %)
Electromyography (EMG) (51 patients)	
Demyelinating sensory motor polyneuropathy	24 (35.3 %)
Sensory Neuropathy	15 (22.06 %)
Axonal sensorimotor neuropathy	4 (5.88 %)
Normal	8 (11.76 %)
Unavailable	17 (25 %)

Otherwise, we noticed that the 56 patients whose diagnosis was not clarified, mostly issued from consanguineous families (39 patients), were represented by 30 males and 26 females (sex ratio of 1.15), a ratio close to the group of 110 patients with genetic diagnosis. The mean age at onset was 7.63 ± 8.17 SD years and ranged from birth to 31 years old, data not statistically different from the subgroup of patients with genetic diagnosis, while cerebellar syndrome, from severe to moderate, was always present. When available, MRI results and EMG examination showed the same heterogeneous features as the group of patients with genetic diagnosis, with no remarkable delineation of particular phenotypic characteristics or additional associated signs. Also, when available, deficiency of vitamin E, hypoalbuminia, or elevated AFP, cholesterol or cholestanol levels, could not be noticed and were in the normal range.

### Molecular characteristics and findings

Among the 110 patients issued from 76 Algerian families, consanguinity was present in 76.36 % of patients (84 patients/56 families) and absent in 18.18 % of patients (20 patients/15 families), whereas an undetermined inbreeding context could be noted for the remaining 5.45 % (6 patients/5 families).

We report 23 different molecular alterations, including 6 not as yet described, in 9 different genes responsible for autosomal recessive cerebellar ataxia forms. In all but one patient, these mutations were found at the homozygous state.

The most common mutation was the (GAA)_n_ expansion in the first intron of the *FXN* (frataxin) gene which is responsible for the Friedreich ataxia phenotype [11], present at the homozygous state in 49 (44.54 %) patients representing 31 (40.79 %) families. 35 of the 49 homozygous patients (22/35 families) belonged to consanguineous families.

In our cohort, 19 patients representing 16 families were found to be homozygous for the c.744delA mutation in the fifth exon of the *TTPA* (tocopherol transfer protein alpha) gene, responsible for ataxia with isolated vitamin E deficiency [12]. 12/19 patients (9/16 families) were issued from consanguineous families.

18 patients representing 12 families were homozygous for different mutations in the *SETX* (senataxin) gene responsible for ataxia with oculomotor apraxia 2 (AOA2). 14/18 patients (9/12 families) were consanguineous.

We also report patients with mutations in the following genes: *SACS* (sacsin), *APTX* (aprataxin), *ADCK3* (aarf domain-containing kinase 3), *ABHD12* (abhydrolase domain-containing protein 12), *SYNE1* (synaptic nuclear envelope protein 1), and *SIL1* (SIL1 nucleotide exchange factor 1), respectively responsible for autosomal recessive spastic ataxia of Charlevoix-Saguenay (ARSACS), ataxia with oculomotor apraxia type 1 (AOA1), spinocerebellar ataxia, autosomal recessive 9 (SCAR9), polyneuropathy, hearing loss, ataxia, retinitis pigmentosa, and cataract syndrome (PHARC), spinocerebellar ataxia, autosomal recessive 8 (SCAR8) and Marinesco-Sjögren syndrome (MSS). These forms were found in less than 10 patients and some of them in a single patient:

- 8 patients belonging to 6 families carried mutations in the *SACS* gene.
- 6 patients belonging to 5 families had mutations in the *APTX* gene.
- 6 patients belonging to 3 families showed mutations in the *ABHD12* gene.
- 1 patient carried a homozygous mutation in the *SYNE1* gene and another patient was homozygous for a mutation in the *SIL1* gene.

Table 3 summarizes the molecular findings for the patients/families for which the mutations were identified.

**Table 3:** Molecular findings in Algerian patients affected with autosomal recessive ataxia

ARCA (Gene)	Number of patients (families)	Family mutation (E, Exon. I, Intron)	Reference
FRDA (*FXN*)	49 (31)	Homo (GAA)_n_ expansion (I1)	[12]
AVED *(TTPA)*	19 (16)	Homo c.744delA ; p.Glu249Asnfs*15 (E5)	[11]
AOA2 (*SETX*)	2 (1)	Homo c.2602C > T; p.Gln868* (E8)	[32]
	1 (1)	Homo c.5267T > C ; p.Phe1756Ser (E8)	[32]
	9 (5)	Homo del exon 17 and 18	[9]
	1 (1)	Homo del exon 5	[9]
	1 (1)	Homo c.5123G > C ; p.Trp1708Ser (E8)	This study
	1 (1)	Homo c.5308_5311delGAGA ; p.Glu1770Ilefs*15 (E9)	[64]
	2 (1)	Homo c.915G >T; p.Trp305Cys (E8)	[32]
	1 (1)	Comp. Heter c.915G > T; p.Trp305Cys (E8) c.985C > T; p.Arg329* (E8)	[32] This study
ARSACS (*SACS*)	1 (1)	Homo c.7372_7376delCTTAT ; p.Leu2458Alafs*5 (E10)	[65]
	2 (1)	Homo c.4882_4886delCAGTT/insAGAAGC p.Gln1628Thrfs*13 (10)	[65]
	4 (3)	Homo c.12220G >C (exon 10), p.Ala4074Pro (E10)	[36]
	1 (1)	Homo c.6355C >T (exon10); p.Arg2119* (E10)	This study
AOA1 (*APTX*)	5 (3)	Homo c.837G >A; p.Trp279* (E6)	[44]
	1 (1)	Homo c.875-1G >A (disruption of splice site) (E7)	[46]
SCAR9 (*ADCK3*)	4 (1)	Homo c.1398 +2T>C; p.Asp420Trpfs*40/ p.Ile467Alafs*22 (I11)	[49]
	1 (1)	Homo c.500_521delinsTTG, p.Gln167leufs*36 ( E3)	[49]
	1 (1)	Homo c.1334_1335delCA ; p.Thr445Argfs*51 (E11)	This study
PHARC (*ABHD12*)	1 (1)	Homo c.846_852dupTAAGAGC; p.His285fs*1 (E9)	[56]
SCAR8 (*SYNE1*)	1 (1)	Homo c.3715G >T ; p.Glu1239* (E30)	This study
MSS (*SIL1*)	1 (1)	Homo c.1285T >G ; p.Tyr429Asp (E11)	This study

*AOA1*, Ataxia with Oculomotor Aparaxia type 1; *AOA2*, Ataxia with Oculomotor Aparaxia type 2; *ARCA*, Autosomal Recessive Cerebellar Ataxia; *ARSACS*, Autosomal Recessive Spastic Ataxia of Cherlevoix-Saguenay; *AVED*, Ataxia with isolated Vitamin E Deficiency; *FRDA*, Friedreich Ataxia; *MSS*, Marinesco-Sjögren syndrome; *PHARC*, Polyneupathy, Hearing loss, Ataxia, Retinitis pigmentosa and Cataract; *SCAR8*, Spinocerebellar Ataxia, Autosomal Recessive 8; *SCAR9*, Spinocerebellar Ataxia, Autosomal Recessive 9; Homo, homozygote; Comp. Heter, compound heterozygte

*Stop codon

## Discussion

The present study is the first description of Algerian patients with autosomal recessive ataxia and represents the largest cohort of patients with genetically characterized ataxia, with 110 Algerian patients (76 families). Table 4 summarizes the clinical features in the patients for the different forms of ARCA studied. Most patients (76.36 %) had consanguineous parents, a proportion that might be higher if we consider distant consanguinity, and all the patients except one carried mutations at a homozygous state, a predictable issue if we consider cultural practices of inbreeding in our population.

**Table 4:** Genotype/phenotype correlations in a group of patients of the Algerian cohort

	FRDA (26P/18 F)	AVED (12P/10 F)	AOA2 (6P/6 F)	ARSACS (8P/6 F)	AOA1 (2P/2 F)	SACR9 (5P/3 F)	PHARC (2P/1 F)	SCAR8 (1P/1 F)	MSS (1P/1 F)
Age at onset (range)	12.74 ± 7.15 SD (4-35)	11 ± 4.69 SD (4-17)	14.57 ± 3.87 SD (9-21)	7.75 ± 4.62 SD (2-14)	3 and 7	9.2 ± 3.7 SD (8-14)	12 and 16	7	birth
Initial signs									
Gait limb ataxia	24	11	6	7	2	5	2	1	/
Dysarthria	/	/	/	/	/	/	/	/	/
Head tremor	1	1	/	/	/	/	/	/	/
Muscle Weakness	1	/	/	/	/	4	/	/	/
Cerebellar Syndrome									
Mild	/	/	/	/	/	5	/	1	/
Moderate	23	10	6	5	2	/	2	/	/
Severe	3	2	6	3	/	/	/	/	1
Head tremor	3	7	/	/	/	1	/	/	/
Dysarthria	23	12	5	7	2	3	1	1	1
Nystagmus	14	7	5	6	/	/	/	/	1
Visual acuity decline	1	1	/	1	/	/	1	/	1
Upper limbs tendon reflexes									
normal	5	2	/	3	/	1	/	1	/
weak	1	/	2	1	2	2	/	/	/
absent	15	10	4	/	/	/	2	/	/
brisk	3	/	/	3	/	1	/	/	1
Lower limbs tendon reflexes									
normal	1	/	/	3	/	1	/	/	/
weak	/	/	2	/	1	2	/	/	/
absent	19	12	4	/	1	/	2	1	/
brisk	4	/	/	4	/	1	/	/	1
Babinki sign	12	4	/	6	/	/	/	/	/
Dysmorphic syndrome									
Scoliosis	19	9	3	1	1	1	/	/	/
Pes cavus	14	7	3	6	/	4	2	1	1
Flat feet	/	3	/	/	/	/	/	/	/
Spasticity	3	1	/	6	/	/	/	/	1
Cognitive impairment	/	/	1	2	2	2	/	/	/
MRI									
normal	10	7	/	1	/	1	1	/	/
cerebellar atrophy	2	1	6	1	1	3	1	/	1
vermian atrophy	1		3	4	1	1	/	/	/
not available	10	4	/	2	/	/	/	1	/
EMG									
Normal	2	4	/	1	1	/	/	/	/
SMP	10	4	3	4	/	/	2	/	/
SN	8	2	3	1	1	/	/	/	/
AN	2	2	/	/	/	/	/	/	/
Cardiac impairment	7	/	/	/	/	/	/	/	/
Epilepsy	/	1	/	2	/	/	/	/	/
Hypoacusia	4	/	/	2	/	/	1	/	/

*AOA1*, Ataxia with Oculomotor Aparaxia type 1; *AOA2*, Ataxia with Oculomotor Aparaxia type 2; *ARCA*, Autosomal Recessive Cerebellar Ataxia; *ARSACS*, Autosomal Recessive Spastic Ataxia of Cherlevoix-Saguenay; *AVED*, Ataxia with isolated Vitamin E Deficiency; *FRDA*, Friedreich Ataxia; *MSS*, Marinesco-Sjögren syndrome; *PHARC*, Polyneupathy, Hearing loss, Ataxia, Retinitis pigmentosa and Cataract; *SCAR8*, Spinocerebellar Ataxia, Autosomal Recessive 8 *SCAR9*, Spinocerebellar Ataxia, Autosomal Recessive 9; /, absence of the sign (no patients)

Our cohort did not include any ataxia telangiectasia patients, although it is reported to be the second most common ARCA [6, 13]. Despite the fact that the gene was included in the panel of genes screened by targeted genomic capture and high-throughput sequencing, no mutations were identified in the Algerian patients. Indeed, since the diagnosis of ataxia telangiectasia is based primarily on clinical criteria in our country, we didn’t get samples to analyze as the evoking phenotype of this disease could be sufficient. Therefore, this form of ataxia could be more common in Algeria.

**Fig. 1:**
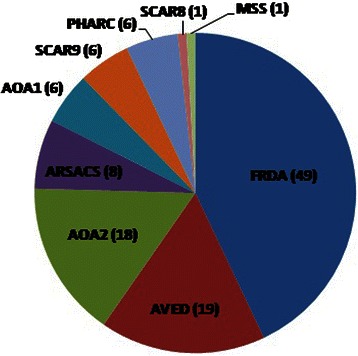
Patients of the Algerian cohort with identified forms of ARCA. AOA1, Ataxia with oculomotor aparaxia type 1; AOA2, Ataxia with oculomotor aparaxia type 2; ARSACS, Autosomal recessive spastic ataxia of Cherlevoix-Saguenay; AVED, Ataxia with isolated vitamin E deficiency; FRDA, Friedreich ataxia; MSS, Marinesco-Sjögren syndrome; PHARC, Polyneupathy, hearing loss, ataxia, retinitis pigmentosa and cataract; SCAR8, Spinocerebellar ataxia, autosomal recessive 8 (or ARCA1); SCAR9, Spinocerebellar ataxia, autosomal recessive 9 (or ARCA2)

However, we were able to report 23 different molecular alterations, including 6 novel mutations, allowing us to identify 9 different forms of autosomal recessive cerebellar ataxias in our population (Fig. 1). Five forms of ARCA appear to be most frequent in the Algerian population.

### FRDA patients

We identified 49 patients (31 families) carrying mutations in the *FXN* gene responsible for Friedreich ataxia (FRDA) [11], in accordance with epidemiological data acknowledging Friedreich ataxia as the most common form of autosomal recessive ataxia in Europe [14]. Even if rare compound heterozygous mutations, (GAA)_n_ expansion associated with a point mutation, were described in some patients, all our patients carried exclusively the homozygous unstable trinucleotide repeat (GAA)_n_ in the first intron of the *FXN* gene, as reported in the majority of patients reported worldwide [15].

In our cohort, 26 of the 49 patients had undergone neurological examination. 24 of them had gait-limb ataxia as a first sign. Head tremor was the first sign in one patient, while another patient displayed muscle weakness.

The age at onset in our cohort ranged from 4 to 35 years old, with a mean onset age of 12.74 ± 7.15 SD years. Most patients (24) had a classical onset age, between 4 and 20 years old, while one patient had onset at 27 and another at 35 years, considering them affected by late onset forms.

Dysarthria, which is considered as a clinical feature in Friedreich patients [16], was present in 23 patients, being severe in two of them.

The upper limb tendon reflexes were abolished in 15 patients, were brisk in 3 and normal in 5. At the same time, lower limb tendon reflexes were abolished in 19 patients, brisk in 4 and normal in one patient. Babinski sign was noted in 12 patients, as pointed out by previous reports [14, 17, 18].

In 22 patients, dysmorphic features was either scoliosis (8 patients), pes cavus (3 patients) or both scoliosis and pes cavus (11 patients), one patient presented with a thoracic deformation, according to the progression of the symptoms in Friedreich ataxia [14].

MRI was mostly normal (10 patients), whereas 2 patients presented with cerebellar atrophy as can be observed in advanced stages of the disease [15, 16]. These patients were diagnosed after 9 and 3 years of disease duration and the age at onset was respectively 20 and 13 years. One of the patients had vermian atrophy, with an age at onset of 4 years and after 20 years of evolution. Another patient showed a parieto-occipital atrophy, with an onset at 27 years and a duration of the disease of 39 years. One patient, with an an age at onset of 14 years and 9 years of disease evolution, showed spinal cord atrophy.

In our cohort, 4 patients (15.4 %) with *FXN* mutations had hypoacusia, as has already been reported in a similar proportion [19, 20].

Electromyography (EMG) was normal in 2 patients, while sensory motor polyneuropathy was found in 10, sensory neuropathy in 8 and axonal neuropathy detected in 2 other patients.

Cardiac impairment, frequent and usually correlated to a premature death in Friedreich ataxia [15, 19, 21], was noted in 7 patients.

None of the patients of our cohort had diabetes, although it has been reported in approximately 10 % of FRDA patients [16].

### AVED patients

The second most common ARCA diagnosed was the ataxia with isolated vitamin E deficiency (AVED), with 19 patients (16 families) genetically confirmed: a unique mutation (c.744delA) in the *TTPA* gene was found at a homozygous state in all the patients, which further confirms its high frequency in the Mediterranen basin [22] and its strong founder effect as suggested by Ouahchi *et al.* [12] and supported by the recent report of El Euch-Fayache *et al.*, 2014 [23] or our personal data (unpublished results).

Among our AVED patients, 12 (10 families) had undergone neurological examination and 8 patients (6 families) were consanguineous.

The mean age at onset was 11 ± 4.69 SD years, ranging from 4 to 17 years old, in accordance with the fact that most of the patients already described have onset before 20 years old [22, 23].

All the patients but one (11) had gait-limb ataxia as a first sign. Head tremor was the initial sign for the remaining patient. Head titubation was noted in 7 out of the 12 patients, a sign that could in fact be considered significant to distinguish FRDA from AVED [23–25].

The upper limbs reflexes were abolished in 10 patients and normal in 2, while the lower limb reflexes were abolished in all the patients. These observations were expected as most AVED patients reported in the literature have absent or weak tendon reflexes [24, 25].

Nystagmus was present in 7 patients and one patient had a visual acuity decline. None of the Algerian patients presented other ocular features such as retinitis pigmentosa [26, 27] or oculomotor apraxia and exotropia [23].

Dysmorphic signs were either solely pes cavus (1 patient) or scoliosis, which was associated with either pes cavus (6 patients) or flat feet (3 patients).

In this cohort, MRI was normal in 7 patients, showed the presence of cerebellar and vermian atrophy in one patient and was not available for 4 patients. EMG results were normal in 4 patients and abnormal in 8: two patients had axonal neuropathy, four patients presented with sensorimotor neuropathy and two patients displayed purely sensory neuropathy. Although AVED is considered to be an ataxia form presenting with a pure sensory neuropathy [14, 15, 28], patients with sensorimotor neuropathy have in fact also been reported, particularly in a Tunisian cohort [23].

All the patients carrying the c.744delA mutation in the *TTPA* gene showed significantly low levels of vitamin E in plasma, as initially stated by this biomarker for this disease [22].

Although it has been reported that an early supplementation with vitamin E can slow down the progression of the disease and maintain the walking capability of the patients [29, 30], most of our patients received an intermittent treatment, due to socioeconomic circumstances, making it difficult to assess the true course of the disease and the real benefit of vitamin E supplementation.

One of our patients that had generalized tonico-clonic seizures presented with early cerebellar syndrome, dysarthria, abolished tendon reflexes, scoliosis and sensorimotor neuropathy at 4 years of age, while cerebral computed tomography was normal, as has been reported for a Norway patient with vitamin E deficiency and epileptic seizures but with compound heterozygous mutations in the *TTPA* gene [31].

### AOA2 patients

The third most frequent form of ataxia in our cohort was the ataxia with oculomotor apraxia type 2 with different mutations in the *SETX* gene. This cohort included 18 patients (12 families) with AOA2, and this study backs up the hypothesis already stated by Tazir *et al.* [9] setting the AOA2 as the third most frequent recessive ataxia in Algeria after FRDA and AVED.

Most of the mutations in our cohort have been previously described (Table 3). One mutation, c.5267 T > C ; p.Phe1756Ser, previously found at a heterozygous state in a patient from the United Kingdom [32] was found in one of our patients at a homozygous state. All patients but one were homozygous for the *SETX* mutations. This patient was a compound heterozygote for a new mutation c.985C > T; p.Arg329* associated with a previously described mutation c.915G > T; Trp305Cys, both in exon eight of the gene [9].

Out of the 18 patients with *SETX* mutations, 10 patients (6 families) have been previously reported [9], while the remaining 8 (6 families) were newly diagnosed patients. Out of the 18 patients, 8 (7 families) were consanguineous, and out of the 8 newly diagnosed patients, 6 (4 families) were consanguineous and 6 (6 families) had undergone a complete neurological examination.

The mean age at onset in our patients was 14.57 ± 3.87 SD years, ranging from 9 to 21 years old, in accordance with the data of other published series of AOA2 patients [9, 33–35].

The 8 newly diagnosed patients presented with cerebellar syndrome, dysarthria and nystagmus, as indicated by the AOA2 phenotype that includes such features in the great majority of the patients [9, 33, 34], whereas only 2 of our patients had oculomotor apraxia, a sign present in less than half of the patients [33, 34].

All patients had elevated alpha-fetoprotein levels which is the main biological marker of AOA2 [32, 33]. Due to the significant size of the gene, only the patients with elevated AFP levels were therefore tested for the presence of mutations in the *SETX* gene. Although pes cavus and scoliosis are considered to be occasional features [9, 33, 34], in our cohort, we could observe 1 aptient with scoliosis only, 2 patients with scoliosis and pes cavus, and 2 patients with pes cavus only.

### ARSACS patients

Our study suggests that ARSACS is the fourth most frequent form of ataxia in Algeria, with 8 patients and 6 families.

We identified 4 different mutations in the *SACS* gene in 8 patients. One of the mutations (c.6355C > T; p.Arg2119*) was novel, and the mutation (c.12220G > C; p.Ala4074Pro), previously described in Tunisian families [36], was the most common mutation and was found in 4 patients (3 families).

All the patients with *SACS* mutations had undergone neurological examination. The 8 patients (6 families) were consanguineous.

The mean age at onset for our patients was 7.75 ± 4.62 SD years, ranging from 2 to 14 years old. Two patients had onset at 12 years old and one at 14 years old, which can be considered as late onset for ARSACS phenotype [36, 37].

Dysarthria was present in 7 of our patients and was acute for one of them, in accordance with one of the major manifestations of the disease during the first two decades of disease progression [37].

Although hyper myelinated retinal fibers has been constantly described in Canadian ARSACS patients [38, 39], we could notice their absence in all Algerian patients, as has also been observed in Tunisian families [36].

Pyramidal signs were observed in our patients: Babinski sign in 6 of our 8 patients; spasticity, either severe or moderate, in 6 patients; upper and lower limbs reflexes, brisk or very brisk, in 4 patients; while tendon reflexes were normal in 3 patients, which could be explained by the progression of peripheral neuropathy that thus mask spasticity and pyramidal signs with the evolution of the disease [37].

Cognitive impairment has been noted for two of the patients of our cohort.

Two patients had epileptic seizures, a feature which has been reported in a small proportion of patients harboring *SACS* mutations [40, 41], while hearing loss, a rare feature not mentioned in ARSACS patients, was found in two of our patients.

A larger study of ARSACS is being conducted in our population and will provide more insights on this ataxia in our population, as the patients from Algeria seems to share clinical phenotypes that can be rather rare and can mislead the diagnosis (such as later onset during the second decade, absence of myelinated retinal fibers, epileptic seizures, mental disability).

The huge size of the *SACS* gene complicates the screening of patients presenting atypical clinical phenotype and indicates that this form of ataxia may be under-diagnosed in several populations, including ours. The screening of the gene is therefore worthwhile even when some features are absent.

### AOA1 patients

The ataxia with oculomotor apraxia type 1 (AOA1) was the fifth most frequent ARCA in our cohort since we have identified 6 patients (3 families) carrying *APTX* mutations, although it seems to be a rare condition outside Japan or Portugal [42, 43].

The nonsense mutation, c.837G > A; p.Trp279*, known as the most frequent mutation in the European population [44, 45], was present in 5 of our patients (2 families), while one patient was homozygous for a splice mutation on the acceptor splice site of exon 7, c.875-1G > A.

A consanguineous context was noted in 5 patients (4 families) and two of these patients (from two unrelated families) had undergone neurological examination.

The age at onset in our patients was 3 and 7 years old respectively, with gait-limb ataxia as a first sign for both patients, accordingly with the early-onset status of this form of ataxia [42, 46, 47].

Our patients had moderate cerebellar syndrome with dysarthria, but absence of nystagmus although this later feature accounts for one of the main clinical signs of AOA1 [42]. However, both patients had oculomotor apraxia and one of them presented with sensory neuropathy. Our two AOA1 patients had cognitive impairment, which was acute for one of them. This feature, initially thought to be exclusive to Japanese patients [43], was nevertheless found in a cohort of Italian patients [47] and is here described for the second time in non-Japanese patients.

MRI showed in our patients a severe cerebellar atrophy in one case and a severe vermian atrophy in the other, as has been reported in all affected AOA1 patients [42, 47, 48].

### SCAR9 patients

The report of a new case of SCAR9 in Algeria in this study, after the identification of the causal gene in two Algerian families [49], brings the number of patients with genetically confirmed SCAR9 to 6 (3 families) in Algeria and to 24 worldwide [49–51].

The patient we report here carried a novel frameshift mutation c.1334_1335delCA, p.Thr445Argfs*51 in the *ADCK3* gene, highlighting that all the mutations identified so far in Algeria are truncating.

Although the delineation of the SCAR9 phenotype remains difficult because of the great clinical differences from one patient to another [51], the clinical features of all the SCAR9 Algerian patients seems relatively similar. In fact, this new patient was diagnosed after 2 years of disease duration and shared with the other Algerian patients already described a similar age at onset (8 years old/mean onset age of 6.8 ± 2.95 SD years, ranging from 4 to 11 years old) and a very mild cerebellar syndrome with gait limb ataxia and muscle weakness as first signs.

The upper and lower limb reflexes were weak for 2 patients including the newly reported one but were either brisk or normal for the others. All patients had dysarthria and pes cavus but only the patient carrying the newly found mutation presented with mild scoliosis. The MRI showed no abnormalities for this patient, which can be explained by the early diagnosis and the short time of disease duration. All the other patients (with 14 to over 30 years of disease duration) showed cerebellar atrophy.

Our patient should also be surveyed for signs of intelectual deficiency as mild forms have been observed in two of the Algerian patients and was reported as mild, moderate or severe in non-Algerian patients [49–52].

All the Algerian patients with SCAR9 seems to have a mild, slowly progressive or even stable cerebellar ataxia, which was suggested as a main feature for SCAR9 phenotype, in contrast with most of childhood-onset ARCA [50–52]. The existence of severe SCAR9 phenotypes can however be observed, probably explained by the occurrence of stroke-like episodes or severe progressive encephalopathy including cerebellar atrophy [51, 53].

### PHARC patients

Our cohort of 6 patients included 2 of the siblings previously described in studies of PHARC and which allowed the identification of the gene, showing that all reported mutations so far results in premature stop codons presumed to cause a loss of function of the protein [54, 55]. All Algerian patients had thus the same homozygous mutation c.852insTAAGAGC (c.846_852dupTAAGAGC); p.His285fs1*. In our study, we screened the *ABHD12* gene for two Algerian patients who presented a phenotype somewhat similar to the Algerian patients identified in the report of Fiskerstrand *et al.* [56]. The two previously reported Algerian patients and included in our cohort had onset at 12 and 18 years of age respectively, had both cerebellar ataxia and peripheral neuropathy; one had an important decline in visual acuity and the other presented with cataract. However, the two new patients we explored did not carry *ABHD12* mutations. Although our criteria were mainly the presence of ataxia and polyneuropathy associated with either cataract, a decline in visual acuity and/or hearing loss, it could not exclude the presence of atypical clinical presentations, as was the case for non-Algerian PHARC patients. Indeed, neither signs of polyneuropathy nor ataxia were observed in these siblings, while their hearing loss and cataract were wrongly attributed to their age or the progression of the retinitis pigmentosa, thereby expanding the phenotypical spectrum of PHARC [55]. We can however assume that this syndrome could be in fact extremely rare.

### SCAR8 patient

We identified in the present cohort a patient carrying a novel mutation, c.3715G > T; p.Glu1239*, in the *SYNE1* gene, thereby reporting the first Algerian patient with a genetically confirmed recessive ataxia SCAR8.

The patient had consanguineous parents and 10 siblings who were all healthy. The age at onset was 7 years old, which can be considered as early-onset in contrast to the findings in the French-Canadian patients who showed a middle-aged onset [57, 58]. The patient had gait limb ataxia as a first sign and presented with mild cerebellar syndrome and dysarthria; she had normal upper limb reflexes but abolished lower ones. The identification of the causal mutation was performed by targeted genomic capture shortly after the first consulting, while MRI and EMG examinations were not done. It would therefore be interesting to see if this patient has a marked diffuse cerebellar atrophy and no signs of neuropathy, as was shown in the French-Canadian patients [57, 59]. The phenotypic features of our patient showed that the outlines of the disease remain ambiguous, with probable great variability in patients outside Quebec and Canada. It also indicates that unrecognized features, such as early onset, may lead to the underdiagnosis of this form of ataxia, particularly because genetic analysis faces such a large gene as *SYNE1* with its 145 coding exons and complicates considerably the mutational screening.

### MSS patient

We report in this study the first genetically confirmed case of Algerian patient with Marinesco-Sjögren syndrome (MSS) and the characterization of a new mutation in the *SIL1* gene, c.1285 T > G; p.Tyr429Asp, at the homozygous state.

This patient had consanguineous parents and had an initial psychomotor impairment that started at birth (sitting position acquired at 2 years and a half, walking without help never acquired, and speech acquisition at 4 years old with an important dysarthria). The patient was 14 years old when diagnosed and had severe cerebellar syndrome (walk without bilateral help was impossible), severe dysarthria, nystagmus and visual acuity decline. The upper and lower limbs reflexes were brisk and the patient presented a mild spasticity but no Babinski sign. Dysmorphic features consisted in pes cavus and craniofacial dysmorphy. MRI showed cerebellar atrophy and hypoplasia. The patient had congenital cataract, but has undergone surgery at 6 years of age. Once the molecular diagnosis established, the patient was also found to have hypergonadotropic hypogonadism, in accordance with the presentation of the syndrome [60, 61].

Although MSS patients have been reported in many countries [60, 62, 63], there is no epidemiological data on the occurrence of this syndrome. Targeted genomic capture and massive parallel sequencing could therefore help to screen for such genes described in rare syndromes.

## Conclusions

We report with this large cohort of genetically determined autosomal recessive ataxia, the first study of the genetic context of ARCA in Algeria. Our study shows that, in addition to the most common mutations and ataxias, other rarer or underdiagnosed forms might be involved and could be underestimated. Taken together, FRDA and AVED patients thus represented 61.82 % of the patients in this cohort (68 patients) while combined, FRDA, AVED, AOA2, ARSACS and AOA1 accounted for 90.9 % of the cohort (100 patients) of the whole autosomal recessive forms of ataxia in our cohort. Besides these most frequent forms, we were able to identify other types of ataxia seemingly rare in our population.

A precise assessment of the frequent forms and mutations specific to our population can strongly help the orientation of the investigations in the long term. However, for rare and heterogeneous pathologies as autosomal recessive ataxia, there is still a long way to go in order to refine genotype/phenotype correlations. More extensive molecular and epidemiological investigations will allow a better knowledge of the different ARCA and facilitate the genetic explorations. The power of innovative approaches, such as high-throughput sequencing, opens now new perspectives for diagnosis and research and will certainly provide mandatory answers for patients remaining without molecular diagnosis.
